# How we can protect the protectors: learning from police officers and staff involved in child sexual abuse and exploitation investigations

**DOI:** 10.3389/fpsyg.2023.1152446

**Published:** 2023-05-10

**Authors:** Theresa Redmond, Paul Conway, Simon Bailey, Peter Lee, Samantha Lundrigan

**Affiliations:** ^1^The Policing Institute for the Eastern Region, Anglia Ruskin University, Chelmsford, United Kingdom; ^2^Department of Psychology, University of Southampton, Southampton, United Kingdom; ^3^Faculty of Creative and Cultural Industries, University of Portsmouth, Portsmouth, United Kingdom

**Keywords:** child sexual abuse and exploitation, mental health and wellbeing, police officers and staff, barriers to support, stigma, workplace and police culture

## Abstract

**Background:**

Police officers and staff who work in child sexual abuse and exploitation (CSAE) investigations are routinely exposed to traumatic materials and situations. Despite support services, working in this space can have negative impacts on wellbeing. This paper explores the experiences and perceptions held by police officers and staff involved in CSAE investigations in the United Kingdom, regarding work-related wellbeing support and barriers to accessing such support.

**Method:**

A sample of 661 serving police officers and staff working in CSAE investigations participated in a United Kingdom-wide ‘Protecting the Protectors’ survey. We analysed quantitative and qualitative responses relating to participants’ experiences and perceptions regarding three main areas: (1) availability, usage and helpfulness of existing work-based well-being support; (2) barriers to accessing support; and (3) desired support services.

**Findings:**

Five interconnected themes emerged from the qualitative data that represented participants’ experiences and views of work-based wellbeing support and the barriers to accessing it. These were ‘Lack of trust’, ‘Stigma’, ‘Organisational approaches to wellbeing’, ‘Support services’, and ‘Internalised barriers’. The findings suggest that whilst respondents were aware of work-based support, they indicated most frequently that they ‘never or almost never’ used them. Respondents also identified barriers to accessing support, which related to a perception of a critical or judgmental workplace culture and indicating a lack of trust in their organisations.

**Conclusion:**

Stigma regarding mental ill health has a pervasive and harmful impact on emotional health and wellbeing of police officers and staff involved in CSAE investigations, which creates a sense of lack of emotional safety. Therefore, eliminating stigma and creating a workplace culture that explicitly values and prioritises the emotional health and wellbeing of the workforce would improve the wellbeing of officers and staff. Police organisations could further improve CSAE teams’ wellbeing by developing a continuum of care which is available to workers from recruitment to the end of the role, training managers and supervisors to better support CSAE teams, improving workplace practices, and ensuring high quality, specialist support services are readily and consistently available across forces.

## Introduction

Police officers and staff who work in child sexual abuse and exploitation^1^ (CSAE) investigations are regularly exposed to traumatic materials and stressful situations. The scope of the tasks and activities that these personnel carry out is broad and varied and can include regularly working directly with child victims of sexual abuse, rape, sexual exploitation, as well as offenders who have perpetrated these offences. They may also work with child sexual abuse materials^2^ (CSAM) such as viewing or categorising images and videos of children being subjected to sexual abuse, reading sexually graphic and abusive grooming materials such as online chats or interview transcripts, and supporting victims, perpetrators, and their families.

Globally, the scale and prevalence of online and contact offences of CSAE is rapidly growing and cannot be overstated. The ‘We Protect Global Threat Assessment’ (WeProtect, 2021, p. 8) states that online child sexual abuse is “one of the most urgent and defining issues of our generation.” For example, in 2020, the National Centre for Missing and Exploited Children (NCMEC) CyberTipline received a “record-breaking” 21.7 million reports of suspected CSAE (O’Donnell, 2021), and 29.3 m items of CSAM were removed from the internet in 2021, another record, representing a 35% increase since 2020 (Hern, 2022). In the United Kingdom, National Crime Agency (National Crime Agency, 2022) reports a substantial increase in online CSAM, rising from 43,072 in 2016 to 113,948 in 2018 and it is estimated that there are between 550,000 and 850,000 individuals in the United Kingdom who pose a sexual risk, online or offline, to children (National Crime Agency, 2021). Childline reports that CSAE is now the most common type of child abuse reported to them they come across (National Society for the Prevention of Cruelty to Children, 2021). In addition, there has also been an increased number of reports of non-recent child sexual abuse, which have required a specialist response such as Operation Hydrant.^3^ The total number of alleged suspects referred to Operation Hydrant is 9,367, and the total number of victims/survivors is 12,701 since 2014 (National Police Chiefs’ Council, 2022). CSAE investigations are conducted within this wider context, and they are often complex and can relate to cases of online contact, current and non-recent CSAE, or a combination of these. The police address the extent of this through a number of different teams or units, such as Policing Online Investigation Teams (POLIT), Internet Child Abuse teams (ICAT), Digital Forensics, and Operational Support. Crucially, CSAE teams work in traumatic and challenging investigations in an ever-growing area of demand.

There is a growing body of research which shows that working in CSAE investigations can carry significant risks to the emotional and psychological health and wellbeing of the police officers and staff involved, and the impacts of CSAE investigations can vary. Some studies recognise the chronic impacts on this particular workforce, such as burnout^4^ (Maslach et al., 2001; Perez et al., 2010; Brady, 2017), compassion fatigue^5^ (Figley, 2002), and moral injury^6^ (Shay, 2014; Lee et al., 2020; Doyle et al., 2021; Tapson et al., 2021). Cartwright and Roach (2020) report that stress, depression and anxiety were the three top reasons respectively, and that police employees took sickness leave between 2008 and 2018. Conway et al. (2023), found that a quarter of CSAE police officers and staff scored in the clinical range for depression and anxiety.

CSAE officers and staff have reported desensitisation, intrusive thoughts, increased hypervigilance, suspiciousness and overprotectiveness (Perez et al., 2010; Powell et al., 2015; Parkes et al., 2018), particularly in relation to their own children (Stewart and Witte, 2020; Tapson et al., 2021). In their development of a traumatic events checklist for police, Miller et al. (2021) asked police officers to rank their work-related traumatic events and found that the most populated category involved children, and included fatalities, abuse and sexual exploitation. Worryingly, some research has highlighted that police officers and staff sometimes feel a need to hide the negative impacts their work is having on them, which stems from an expectation that they should be able to cope (Parkes et al., 2018; Foley et al., 2022). This is perhaps indicative of negative workplace and societal attitudes towards mental health and wellbeing.

More serious and potentially acute impacts have been reported by CSAE investigators. Some research suggests that one in five police officers in general suffer with Post-Traumatic Stress Disorder^7^ (PTSD) or Complex Post-Traumatic Stress Disorder (CPTSD) (Miller et al., 2021) resulting from direct exposure to trauma. Hurrel et al. (2018) found that 35% of their sample of CSAE investigators (*n* = 101) would have met the criteria for PTSD; however, not all research in this area supports this: for instance, Conway et al. (2023), found that 3.3% of their sample (*n* = 661) met the criteria for clinical levels of PTSD and 5.4% met the criteria for C-PTSD. Research investigating the prevalence of secondary traumatic stress^8^ (STS) (Figley, 1995), found that just over a third of their sample (*n* = 28) of investigators of online CSAE experienced moderate to high levels of STS (Perez et al., 2010), whilst MacEachern et al. (2019) report that half of their sample of detectives (*n* = 63) from Family Protection Units experienced some degree of STS symptoms.

Recent years have seen a growing recognition within policing for the need to safeguard the emotional and psychological health and wellbeing of police staff and officers generally (College of Policing, 2020a; Oscar Kilo, 2023) and there is now explicit acknowledgement of the impact of exposure to work-based trauma materials and situations, and the need to manage trauma risk (Hesketh and Tehrani, 2018; College of Policing, 2020b). Whilst these resources may be of use to CSAE officers and staff, there is currently only one College of Policing guidance document that is specific to CSAE teams (College of Policing, 2019). It is evident that mental health support is available for CSAE police officers and staff in the United Kingdom (Hesketh and Tehrani, 2018; Conway et al., 2023), and some research suggests that good support can mitigate the negative impacts of working in this space (Tehrani, 2016; Foley et al., 2022; Conway et al., 2023), but it is also evident that there are significant barriers which prevent CSAE officers from seeking mental health support.

Negative impacts on wellbeing are also the result of chronic work-related stress stemming from organisational factors such as low staffing levels, budget cuts, poor leadership and support within any workplace, including policing (Sollie et al., 2017; Deschênes et al., 2018; Brown, 2021), and so it is important to note that workplace culture and wellbeing have a symbiotic relationship. As such they have to be considered together within the wider socio-cultural context, particularly regarding the stigma attached to mental ill health. The relationship between stigma^9^ towards mental health and police culture, whereby ideas about archetypal masculinity can dominate (Evans et al., 2013; Tapson et al., 2021), has been acknowledged as problematic in relation to officer wellbeing (Karaffa and Koch, 2016; Velazquez and Hernandez, 2019; Burns and Buchanan, 2020; Brown, 2021). Furthermore, where they intersect has been recognised as creating barriers to seeking mental health support amongst police officers (Velazquez and Hernandez, 2019; Edwards and Kotera, 2021; Newell et al., 2022). Some research highlights ongoing concerns regarding barriers to accessing support (Doyle et al., 2021) and the efficacy of support that is provided to CSAE investigators (MacEachern et al., 2019).

Police and workplace culture can shape organisational and personal responses to mental health issues and consequently can have a powerful influence on wellbeing, access to, and quality of, support services. Whilst some research has highlighted positive impacts of police culture which includes a sense of belonging and loyalty (Alexander and Wells, 1991), other research has shown how police culture can cause harm by minimising or denying the importance of recognising mental health struggles, and silencing those who are struggling (Hetherington, 1993). For example, officers reported to Evans et al. (2013) that talking about personal mental health struggles was perceived as ‘risky’, and there were concerns about being perceived as ‘weak’. Officers and staff who experience this kind of workplace are likely to find seeking support more challenging (Burns and Buchanan, 2020). Velazquez and Hernandez (2019, p. 717), in their research on police officer mental health, discuss how mental health stigma within policing can often be “endorsed.” Karaffa and Koch (2016), in their study exploring stigma and attitudes to seeking mental health support amongst police officers (*N* = 248) in Texas, found that officers who perceived that the general public’s attitude towards mental health as negative were more likely to hold negative attitudes towards seeking professional mental health support. Carleton et al. (2019), in a study exploring the attitudes of public safety personnel (which included police officers) towards mental health support found that 45–60% would only seek mental health support as a last resort.

The impacts of the traumatic content of CSAE investigations, exacerbated and compounded by an unsupportive culture towards wellbeing, can damage the resilience of the individual and this particular workforce (Perez et al., 2010; Brady, 2017; Sollie et al., 2017). For example, the Police Federation (2022) reported that in the past financial year (2021–2022) 13,263 police officers were absent from work due to depression, anxiety, stress or post-traumatic stress disorder (PTSD), a rise of 4,813 from the previous year. Cartwright and Roach (2020) analysed sickness absence data for the last 10 years from 20 United Kingdom police forces and found police absence as a result of mental health conditions has almost doubled, giving an absence rate of 8.82%. The negative impacts of the Covid-19 pandemic on policing, which is beyond the scope of this paper, have also been recognised as exacerbating stresses and pressures on police officers and staff (Stogner et al., 2020; Tehrani, 2022).

CSAE investigators and teams are facing increasing and changing demands, particularly relating to the scale and prevalence of online and contact CSAE. As CSAE offences continue to grow, so too do the operational and psychological demands on this particular workforce. However, a resilient workforce is needed to effectively meet these challenges and access to appropriate and effective support provision is essential to support wellbeing, and this involves acknowledging, tackling and removing barriers that prevent access to good quality support services. It is clear that although support for CSAE police officers and staff is available (Conway et al., 2023; Oscar Kilo, 2023), there is a paucity of research that focuses on CSAE investigators’ experiences and perceptions of work-based wellbeing support, what they identify as barriers to accessing such support and the support services they would like.

The aim of this paper, therefore, is to present findings from the ‘Protecting the Protectors’ survey (Conway et al., 2023) relating specifically to the experiences and perceptions of police officers and staff involved in CSAE investigations in the United Kingdom of existing work-based wellbeing support, the barriers to accessing support, and desired support services.

## Materials and methods

### The ‘Protect the Protectors’ survey

The ‘Protecting the Protectors’ survey was an anonymous online survey designed by the authors to investigate the mental health and wellbeing of police officers and staff involved in CSAE investigations. The survey was open to all serving police officers and staff routinely involved in CSAE investigations across England, Wales, Northern Ireland, and Scotland. The survey was compiled using the online survey tool Qualtrics and open between February 2022 and August 2022. The survey collected quantitative and qualitative information on participants’ demographic characteristics; contact with CSAE victims and perpetrators, exposure to CSAE material, and support factors; mental health and wellbeing; and experiences and perceptions of work-based well-being support and the barriers to accessing such support. Survey questions and scales were developed from a review of the relevant literature. A write-up of the survey results outside of the scope of the present paper can be found in Conway et al. (2023).^10^

### Recruitment

The National Police Chief’s Council (NPCC) lead for Child Protection and Abuse Investigations in England and Wales endorsed the survey and acted as a gatekeeper for the research. An email outlining the nature and purpose of the research, including the link to the online survey, and details of an incentive to participate (i.e., being entered into a draw to win one of 20 £25.00 Amazon vouchers, on completion of the survey) was disseminated through all 43 police forces in England and Wales inviting officers and staff of any rank, who routinely work in any CSAE investigations, in any team or unit to take part in this research. The survey was subsequently extended to include forces from Scotland and Northern Ireland, utilising a similar recruitment strategy.

### Sample

A total of 1,056 surveys were submitted online. Of these, 37% (*n* = 395) were excluded for several reasons (i.e., refused consent to their data being included, *n* = 15; completed <50% of the study, *n* = 156,); completed the study in <200 s with few responses, *n* = 38, failed an attention check (Oppenheimer et al., 2009) question, *n* = 186. The final sample consisted of 661 participants: 71% police officers and 29% police staff. All participants reported that they were currently active members of United Kingdom police forces or police staff who routinely and regularly deal with CSAE, except for 18 people who either recently retired or had been recently removed from CSAE investigations. We nonetheless retained these participants due to their recent service.

Of the final sample, 43% of participants were male, and 57% were female. The average age was 40.61 years (SD = 9.15). The majority (97.1%) identified as White, British, or Caucasian. Participants served in all regions of England (86.9%), Wales (2.4%), Scotland (6.5%), and Northern Ireland (4.2%). Most participants reported working more than a year as a police officer or staff, with an average service length of 10–14 years on a scale from 1 (*only a few days*) to 9 (*over 20 years*; *M* = 7.21, SD = 1.54). Most participants reported working at least a year in their current role, with an average service length of over 2 years (*M* = 4.77, SD = 1.25, on the same 9-point scale). Eighty percent of participants reported having roles in the Child Protection Unit, Investigations, or Police Online Investigation Team, though a minority reported a wide variety of other roles, such as Intelligence, Management of Sexual or Violent Offenders, Operational and Administrative Support, and Neighbourhood Policing.

### Data

The quantitative data used in the current paper were drawn from five of the ‘Protecting the Protector’ survey questions. The questions focused on participants’ experiences and perceptions of existing work-based well-being support; perceived barriers to accessing such support, and their desired support services. Table 1 provides an overview of the questions and response options from which the quantitative data was derived. Qualitative data was derived from collective free text responses provided by participants. Three opportunities to provide additional comments were given to participants during the survey: 50 comments were made in relation to current provision; 95 comments in relation to barriers; and 70 comments in relation to desired resources.

**Table 1:** Overview of quantitative survey questions and possible response options.

Question	Response options
What mental health and wellbeing or personal support does your force currently provide? Seven options given	Tick all that apply from seven possible options: Occupational Health Therapist; external/Self-referred counselling, peer support program, mental health or wellbeing days off, Clinical Supervision, Oscar Kilo [Police Counselling Service], wellbeing of Investigators Toolkit
How often have you used each mental health or wellbeing resource?	Rating scale from 1 (*almost never*) to 7 (*almost always*)
How helpful or useful have found each resource?	Rating scale from 1 (*very unhelpful*) to 7 (*very helpful*)
Thinking about possible barriers that might stop you from asking for mental health or wellbeing support at work, how much is each of the following a barrier for you?	Seven potential barriers provided with a rating scale from 1 (*Not at all*) to 7 (*A large amount*) I feel pressured to seem strong in front of colleagues; Workplace culture where weakness seems not allowed; Wanting to seem capable of handling stress and performing well; It seems like everyone else can cope with this job; Worried that getting support might undermine my job prospects; I’m not convinced that seeking support will help and it might even cause problems; I do not trust my organisation to keep my support confidential
What support do you wish was available?	Tick all that apply from 14 possible options: Monthly group sessions with team; Monthly wellness check-in; 24/7 access to support; Limiting daily exposure to child sexual abuse materials; Separating viewing online CSAE tasks and interviewing victims and/or perpetrators; A workplace culture that explicitly values and prioritises the emotional health and wellbeing of the workforce; A wellness room; Informal peer support; Wellness events (workshops, training); Wellness plans; Social activities; Mindfulness sessions; Clinical supervision; Self-referral to funded counselling (separate from work)

### Analysis

Descriptive statistics for the quantitative data were calculated using SPSS and Microsoft Excel. Qualitative data were derived from responses to the three open-text questions, which ranged from short statements to several sentences. The organisation of the data was pre-determined by the responses to the five survey questions (see Table 1), and this provided a framework for the analysis. Thematic analysis (Braun and Clarke, 2006) was used to code the data and identify themes, within and across this framework, which were identified by one researcher and cross-checked by a second. After familiarisation with the dataset, the data were coded, and semantic themes^11^ were developed, reviewed, and defined. Data were selected, in the form of quotations, to illustrate the thematic findings and foreground the experiences of the participants in order to learn from them (Castleberry and Nolen, 2018).

### Ethics

Research ethics approval for this study was granted by the University of Portsmouth Faculty of Creative and Cultural Industries Research Ethics Committee, Reference Number CCI-FEthC 2022-003, on 14 February 2022. This initial research ethics approval was for a survey of CSAE police officers and staff across all England and Wales constabularies. On 9 May 2022 and 29 June 2022 minor amendments to the original research ethics protocol were requested – and approved – to extend the study to CSAE police officers and staff in Police Scotland and the Police Service of Northern Ireland, respectively.

## Findings

Findings are presented in three sections. Firstly, the quantitative findings concerning current work-based wellbeing provision are presented. Secondly, the quantitative and qualitative findings pertaining to barriers to accessing wellbeing support at work are presented. Thirdly, the quantitative and qualitative findings relating to desired support services are presented.

### Current work-based wellbeing provision

Respondents reported accessing a range of different wellbeing supports services. Table 2 provides an overview of the findings relating to the availability, usage and perceived helpfulness of seven different types of work-based wellbeing support.

**Table 2:** Overview of the availability, usage, and helpfulness of work-based wellbeing support.

Resource	Availability (Yes/no)		Usage (1 – almost never to 7 – almost always)		Helpfulness (1-very unhelpful to 7 – very helpful)	
	*N*	Percent	*M*	SD	*M*	SD
Occupational health therapist	535	80.9	2.11	1.43	3.90	1.91
External/self-referred counselling	395	59.8	1.94	1.47	4.53	2.13
Peer-support program	304	46.0	1.32	0.96	3.46	2.20
Mental health or wellbeing days off	118	17.9	1.31	0.98	3.72	2.45
Clinical supervision	33	5.0	1.16	0.82	2.22	1.89
Oscar kilo^1^	239	36.2	1.17	0.72	2.64	1.98
Wellbeing of investigators toolkit^2^	166	25.1	1.22	0.78	2.95	1.98
Other	78	11.8	–	–	–	–

1 This is the national wellbeing service for police officers in the United Kingdom.

2 Developed as a wellbeing resource by Oscar Kilo.

In relation to availability, the most common type of support was an occupational health therapist with ~80% of respondents reporting access to such a service through their organisation, followed by external self-referred counselling (~60%). Only these two services were reported as available to over 50% of the participants. The least available support service was clinical supervision, with only 5% of respondents reporting access.

Participants were then asked to report the frequency with which they used the support resources provided by their force on scales from 1 (*never or almost never*) to 7 (*always or almost always*). As indicated by the low mean values in Table 2, participants indicated most frequently that they ‘Never or almost never’ used the available supports. Conversely, respondents indicated least frequently that they ‘Always or almost always’ used support. Participants also reported how helpful they had found a resource. ‘External/Self-referred counselling’ was the most positively rated service with a mean score of 4.53 (SD = 2.13). The ‘Clinical Supervision’ and ‘Oscar Kilo’ services were rated the least helpful with mean scores of 2.22 and 2.64, respectively.

### Barriers to accessing wellbeing support at work

**Figure 1:**
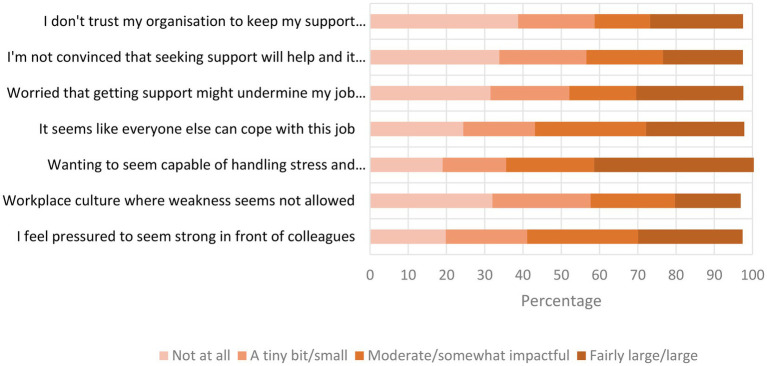
Barriers to accessing wellbeing support at work. Responses do not add up to 100% as not all participants responded to each option.

Respondents reported a number of barriers to accessing wellbeing support in the workplace. Figure 1 illustrates the extent to which participants rated seven different potential barriers as preventing them from accessing wellbeing support at work. Mean ratings and standard deviations are given in brackets. ‘Wanting to seem capable of handling stress and performing well’ was perceived as the greatest barrier to accessing support with the highest mean rating (*M* = 4.63, SD = 2.03), only 17% of participants did not see this as a barrier at all and 42% rated it at the highest end of the scale. The barrier with the lowest mean rating was given to ‘workplace culture where weakness seems not allowed’ (*M* = 3.16, SD = 2.04).

Respondents were provided with the opportunity to offer their views regarding possible barriers to seeking support. Five interconnected themes emerged from this data that represented participants’ experiences and views of work-based wellbeing support: ‘Lack of trust’, ‘Stigma’, ‘Organisational approaches to wellbeing’, ‘Support services’, and ‘Internal barriers’. These themes overlap and interact with each other: for example, a sense of mistrust in the organisation’s approach to wellbeing can be reinforced by a negative experience or perception of a support service, or vice versa, which can influence individuals’ willingness to engage, all of which can then impact their sense of wellbeing.

### Lack of trust

‘Lack of trust’ was omnipresent across the data and permeated through all the other themes, suggesting this was a significant barrier for the participants in relation to accessing wellbeing support. Woven through many of the participants’ responses was a palpable sense of mistrust, and an explicit lack of trust in their organisation’s approach to wellbeing in terms of their organisation’s culture, policies and practices, and the nature and quality of the wellbeing support services they offered. This lack of trust exacerbated a lack of confidence towards an organisation’s ability to support their wellbeing. Some responses had an emotional undertone and expressed participants’ cynicism, anger, and resentment:

[I] do not trust any Force led support

Lack of confidence in the current system, having used it for nearly 10 years.

I do not trust the organisation, there is a rotten culture in senior officers who only consider their own prospects and career … why would you trust the most deepest [sic] troubling thoughts with these narcissists who caused them in the first place.

Participants perceived that wellbeing support was often “*minimal and poor*,” given ‘lip-service’ as a “*a box ticking exercise*” instead of being embedded in a system that proactively cares for and supports staff. This perception perpetuated a lack of trust and led to staff feeling uncared for and under-valued:

I don’t feel any trust in the organisation. I feel like all the talk about mental health and wellbeing is a PR stunt and political tool within the workplace. I genuinely do not feel that nobody [sic] cares about mental health or wellbeing in the workplace.

I feel my employer sees mental health support as a box to tick and have no real desire to help individuals when the process raises difficult scenarios.

### Stigma

Reference to stigma around mental ill health, both explicit and implicit, was a recurrent theme In participants’ comments, and shares a symbiotic relationship with ‘lack of trust’. Responses that related to stigma included references to being “*embarrassed*,” having a “*fear of judgement*,” and an awareness that any distress they may be experiencing could be perceived as a weakness or inability to cope with their job. Participants reported feeling concerned about stigma negatively shaping other people’s perceptions of their ability to do their job, being negatively labelled or judged, and damaging their future job prospects and opportunities. The feelings encapsulated In these responses closely relate to the ‘lack of trust’ theme.

Culturally, there is still a massive stigma attached to mental health, and [I] have even heard officers and supervisors within our role discuss the difficulties/(im)possibilities of getting a job within the organisation once diagnosed with MH or being signed off with stress.

I was once told that my file had an entry saying I ‘lacked emotional strength’. I only found out about the entry when it was used in an interview as a reason I would not be successful.

One participant shared an experience whereby they had to take several months off work due to poor mental health and when they returned to work they were “*shipped off to a back office to input figures into a computer while they figure out where to put me! None of my skills or talents are being put to use and it makes me feel demoralised.*”

Stigma and judgements associated with mental health and wellbeing also impacted on how likely or comfortable some participants felt about using some of the resources or strategies available to them:

I believe exercise helps relieves stress. Being able to go for a run/walk/gym during the day and not being judged for it.

a day off for when you feel everything is too much, without being judged or made fun of

### Organisational approaches towards wellbeing

This theme relates to participants’ perceptions of their organisation’s culture, work practices, and approaches to the wellbeing of its officers and staff. Participants perceived this in terms of investment in wellbeing evidenced by the availability and the quality of support services. Within this theme three subthemes emerged: ‘Workplace cultures’, ‘Managerial support’ and ‘Confidentiality’, which particularly relate to the ‘Lack of trust’ and Stigma’ themes and illustrate how all these issues can work together to negatively impact wellbeing.

#### Workplace cultures

The first subtheme of ‘Organisational approaches towards wellbeing’ refers to unsupportive workplace cultures regarding wellbeing, and was identified by participants as problematic and in need of change and improvement:

I think the culture needs to change. Mental health struggles and being signed off work etc is still a taboo and is frowned upon. It is definitely seen as a sign of weakness – there is a, ‘other members of staff are coping and always have done, then why aren’t you’, kind of attitude. It is seen as an occupational hazard, which we all need to accept because we have decided to [do] these types of roles.

I think a cultural barrier still exists, i.e., ‘Police Officers just get on with it’.

Comments indicated negative and critical perceptions of their force’s approach to wellbeing, highlighting a failure to prioritise wellbeing:

Support does not seem a priority by the organisation. It is target focused to the detriment of staff well-being.

Lack of funding and resources from the organisation to provide the necessary wellbeing/mental health support to a team dealing with CSE. Current wellbeing support is insufficient, and the organisation does not appear to be invested in it.

Some comments illustrated minimising and dismissive processes regarding wellbeing within their workplace; for example, the expectation that they should ‘just get on with it’ and the sense that wellbeing is not taken seriously or even acknowledged. Responses also indicated that some participants feel disposable and even dehumanised by the approach to wellbeing in their organisation.

I strongly feel that a lot of the ‘it’s ok not to be ok’ is a load of rubbish. It is only OK if it suits the organisation. I have heard of dozens of examples where people have been ignored because them being ‘not ok’ does not fit with the unit that they are working in.

We are treated as just a number and not an individual, they will work you into the ground until you are burnt out. It is more about stats than people’s welfare.

There was a further recognition by participants that wellbeing support can focus more on capability issues than wellbeing and support, which can also impact on current and future job prospects:

The 6-month psychological appointments seem to focus entirely on my capability of doing my job. This results in concern surrounding seeking support, because I think it will be used against me to tell me I can no longer do my role.

You may be gated from the role, so staff are less likely to use this.

Other comments pointed out that “*prevention should be at the forefront*” of wellbeing provision, but support was perceived by participants as being more reactive, meaning that staff’s wellbeing may deteriorate due to a lack of preventative support:

Lack of knowledge from supervisors to support that is available or lack of knowledge to spot the signs when team members are struggling. Only ever pick it up when it is all too late, and the individual is already broken.

… there was a mental health survey that came out that ignored people who requested to see someone. The next year the option to see someone was removed from the survey. The survey has not been seen since.

Participants identified lack of time as “*a massive barrier*” to seeking help or taking time off work to recover. Others highlighted the combination of a lack of time and high workloads working together as a barrier to seeking support and to taking the breaks they may need to protect their mental health, illustrating that support that involves taking time out is not necessarily a solution:

Time. Work is so busy that to take time out to get support means less time to do the work – vicious circle.

Case Load will be increased whilst away seeking support for mental health.

Time and admin – I am busy all day at work and have a family at home, it is difficult to find the time to complete the admin. Nobody does your work while you are away.

#### Managerial support

The second subtheme of ‘Organisational approaches to wellbeing’ relates to participants’ perceptions of the nature of managerial support. Central to this was criticism of team leaders and/or senior managers and barriers that were perceived as coming from, or being exacerbated by, them. Criticisms ranged from requests for help being blocked or ignored by senior managers to a lack of knowledge, understanding or interest in staff wellbeing. It may be that these experiences and perceptions serve to erode any existing trust and perpetuate mistrust.

It also feels like there are management barriers to assistance, with attempts to change being ignored or blocked by senior management who instead of offering support want another form filled in … We have also had instances where proposals for improving access to mental health services have been ignored by the same member of management …

Everyone wants to think the issue is with seeing images and dealing with scenes, but mainly is it poor management and lack of staff so there is no respite.

A dismissive comment from a manager can have a bigger impact than the actual job content!

#### Confidentiality

The third subtheme of ‘Organisational approaches towards wellbeing’ was central to the permeating theme of ‘Lack of trust’. Participants’ responses revealed concern about whether confidentiality regarding their wellbeing would be maintained by their supervisor, team, and force. Many participants expressed concerns about what they would and would not share because “*it feels like nothing is kept confidential in regard to mental health, and that it is still frowned upon*.”

I would not talk to my supervisor about my mental health – the whole team would be made aware!

There have been several incidents recently where colleagues I know have gone to Fed Reps and Supervisors for confidential support only to have the very same issues gossiped about and shared publicly. I would not trust that this would be any different if I were to seek support.

Nothing is confidential in the police

This organisation definitely does not keep anything confidential!

The comments suggest that some participants do not feel safe to share any mental health issues they experience because it may be gossiped about, shared with other team members and/or held against them, leading some staff to feel like they need to protect themselves from the organisation. This sentiment was reflected in some comments regarding police wellbeing resources such as the ‘Blue Light Champions’ service in relation to peer support. Participants described this service as “*lip service*” and expressed concerns that it does not offer support specific to CSAE teams.

### Barriers to support services

This theme encapsulates participants’ comments regarding the available support services. Participants identified barriers regarding the access to, and quality of, available support services that prevented them seeking support for their wellbeing. Many of the comments referred to a lack of specialist support, referrals to services, long waiting times, and service provision, which was impersonal, unreliable, and inconsistent.

#### Access to support services

Participant’s decision whether to access support or not was influenced by other factors, such as lack of trust, concerns regarding confidentiality and the potential negative consequences disclosing mental health issues could have on them. This participant’s comment illustrates this

A lot of my concerns about seeking help is the concern that my personal details would not be confidential, or that they would be stored indefinitely in HR and used as an excuse (either overtly or quietly) to hamper my future job prospects. I realise that this may sound a bit paranoid, but I’ve experienced similar things within the organisation previously and now try to protect myself where I can.

Participants highlighted that self-referral to support services was a barrier and could be challenging if someone was struggling with their mental health.

I have seen how the job has treated people with mental health issues in the past. People need to self-refer and if they are in mental distress it may be hard to know what the issue is. Due to distrust with how the organisation would handle it I self-referred via the NHS instead of taking advantage of internal mental health support.

Others identified referrals through supervisors as a barrier:

To access support through work, the referral has to be put in by a supervisor, you cannot self-refer. This is a huge barrier for a lot of people.

Participants saw mandatory wellbeing sessions as a way to address concerns around self, or supervisor, referral (this is discussed below in ‘Desired Resources).

One participant shared how they have sought help too late as a result of not knowing what support was available which suggests a lack of appropriate information and signposting:

Personally, I have only sought help after the horse has bolted and really have no knowledge of what is available or out there prior to the horse bolting.

#### Quality of the support services

Of those who had experienced support services, comments were critical of the long waiting times and a lack of reliability within the Occupational Heath Therapist (OHT) service; for example, “*months long waiting list with appointments cancelled at short notice on a regular basis*.” Some responses suggest noticeable inconsistencies in the frequency of provision and availability of OHT sessions, with some participants indicating that appointments can be 6 or 12 monthly. In one case the provision of the service does not meet the requirements of policy: “*We are supposed to have a yearly assessment, however in my 3 years this has never happened*.”

One participant shared that after self-referring to an external counselling service, they still had not heard back from the service since “*over a month ago*,” and another was offered external counselling but was told that “*due to the nature of my work an internal counsellor would be better*,” highlighting the particular issues intrinsic to CSAE work. Concern was also expressed about how the service is often provided over the telephone, which one participant felt “*does not help one bit as you really need a face-to-face meeting with someone to fully understand their emotions and thoughts*.” This was echoed by other comments highlighting how the impersonal nature of some services could be a barrier when sharing personal experiences regarding mental and health and wellbeing:

An annual phone call ticking boxes set by the senior leadership team, not about my welfare.

This is an email once a year to complete a questionnaire. This is then followed up with a telephone call.

Some participants’ comments highlighted a lack of specialist training to support individuals in this area of work:

In my experience, Occupational Health have no idea how to engage with those working in these kinds of arenas, are either disinterested or actively try and avoid speaking about the crime type

We have occupational health but no trained therapists.

Participants reported that OHT was unable to offer them support them because they were ‘too’ emotionally unwell:

[I was] contacted by OHU [OHT] due to my Burnout score. They told me there is nothing to help me.

But my depression has been too severe to qualify for support

Some participants shared experiences of utilising and trusting some support services but almost coming to regret this. One participant shared their experience:

I have engaged with my Force’s occupational health Mandatory Mental Health Assessment process for a number of years culminating in being marked as “at risk” over 6 months ago. This has resulted in no positive action to address the concerns that I, and other colleagues in my department raised… I feel my employers see mental health support as a box to tick and have no real desire to help individuals when the process raises difficult scenarios.

This participant highlights that they are “marked” as ‘at risk’ yet in the following 6 months did not receive any further support. This example encapsulates similar experiences reported by participants and provides an example of how trust in the services and in their force can be undermined and eroded.

### Internalised barriers

This theme offers a more personal perspective and draws from the more personal reasons participants gave for choosing not to seek help. This is no way to suggest that these individuals are in some way to blame for not seeking support rather it gives an insight into some of the ways in which they perceived themselves and how they made sense of seeking support within their organisational context For example, one participant said that they were concerned that “*digging up the past may cause a problem that is not currently there*” and conversely, another expressed a “*fear of opening “the box” in my head.*” A sense of reluctance to seek support, often linking to a lack of trust, was woven through many of these comments.

… whenever I express that I am stressed, I feel like it is held against me … As a result, I am now very reluctant to share any of my feelings/well-being etc.

Other participants recognised that their personality played a role in their decision, and reluctance, to seek wellbeing support:

Part of my personality is to ‘bottle up’ my emotions and deal with things on my own. This is a hard habit to break to be able to seek help.

Personal barriers/stubbornness.

Some participants compared themselves with how other people *appeared* to cope and questioned “*so why could not I?*.” One participant illustrated how they felt “*like other people are more worthy of help*” than themselves. Participants did not seek support because they were aware of the impact this could have on colleagues, especially mental health professionals who are unfamiliar with CSAE:

… being concerned that the supervisors have enough on their plate without me adding to it.

Do not want to cause more stress to my colleagues.

Lack of specialists who understand the nature of the work and fear of causing secondary trauma to them by discussing specific issues caused by the experiences faced.

Comments suggest a sense of disengagement regarding the effectiveness of work-based wellbeing support and some participants expressed that they would not turn to, or rely on, work-based supports. Several such comments came from people who had had a negative experience of work-based supports. For example one participant, after being identified as at risk but not receiving any further help or support, decided to ‘go it alone’ in order to safeguard their mental health:

I have moved on from this [work-based supports] and choose to safeguard my mental health in ways I know help me, such as socialising, running and cycling.

Comments also indicate that some participants felt demoralised by their working conditions and the wellbeing support on offer to them, expressing a sense of pointlessness due to a lack of solutions:

There is no solution to the cause of the stress i.e. too few officers and too much work. it will make no difference. I will share my stresses and anxiety and will go back to work the next day into the same stresses and anxiety.

I also feel that much of the time there is no solution to “make it right”. I think it is more about devising strategies to manage on a day-to-day basis.

We are constantly told that there are not enough people in occupational health, therefore why bother?

I am worried that if I speak honestly about how much I struggle with my workload that I will be asked to go off sick with stress. I feel that being at home/off work thinking about my work mounting up in my absence would cause me even more stress.

### Desired support services

Table 3 shows the extent to which participants would like their force to offer a range of support services. Participants were able to select multiple responses to this question. ‘A workplace culture that explicitly values and prioritises the emotional health and wellbeing of the workforce’ was the most highly rated support option (60.5% of participants), closely followed by ‘social activities’ (58.7%). ‘Self-referral to funded counselling (separate from work)’ (49.3%), ‘Monthly wellness check-in’ (48.4%), and ‘24/7 access to support’ (47.5%), attracted endorsements from nearly half of the participants.

**Table 3:** Desired support services.

Resource	*N*	Percent
A workplace culture that explicitly values and priorities the emotional health and wellbeing of the workforce	400	60.5
Social activities	388	58.7
Self-referral to funded counselling (separate from work)	326	49.3
Monthly wellness check-in	320	48.4
24/7 access to support	314	47.5
Mindfulness sessions	271	41.0
Wellness events (workshops, training)	262	39.6
A wellness room	248	37.5
Monthly group sessions with team	222	33.6
Limiting daily exposure to child sexual abuse materials	194	29.3
Informal peer support	186	28.1
Wellness plans	151	22.8
Clinical supervision	145	21.9
Separating viewing online CSAE tasks and interviewing victims and/or perpetrators	88	13.3

### Improved workplace culture and practices

Participants’ additional views about other wellbeing resources they would find helpful indicated they wanted an improved workplace culture and improved practices to better support and protect their mental health and wellbeing. The comments are largely in line with the findings illustrated in Table 3.

#### Improved cultures

Workplace culture refers to participants’ desire for a “*a culture change whereby senior officers and managers listen and believe the health and wellbeing concerns of their staff and explore how to address them.*” There was also an appetite for the onus of support services to be on the prevention of the deterioration of wellbeing “*before it’s too late and it is necessary to go to occupational health*”:

Mandatory one to one sessions with a qualified professional who is capable of picking up problems with Officers before they get so bad they go off sick.

Other comments gave more detail as to what a supportive workplace culture could look like to participants, specifically in relation to senior officers and managers:

Actually, listening to staff and attempting to lower the demand on individual officers and realising when there are patterns in behaviour, such as whole departments feeling stressed and overworked

There was also a recognition of the need to normalise the experience of struggling with the impacts of this work and accessing the necessary support services and strategies, and some comments highlighted the role that senior officers could take to support this:

A wellness room would be helpful providing it is not frowned upon or seen as a weakness if you decide to use it. I would currently like to use a wellness room but would never do it due to this attitude. Having supervisors encourage this or seeing supervisors use it themselves would be helpful.

Encouraging officers to take breaks, there is culture within certain areas of the policing to not take breaks

#### Improved practices

Participants gave examples of ways to increase wellbeing and access to support services by improving workplace practices. Several comments highlighted the desire for preventative improvements, such as reducing workloads and better training for supervisors. One participant summed this up as, “*more staff and better management*.” Others gave more detailed and specific suggestions to improve work practices such as: limiting caseloads and time spent in CSAE units; reducing single crew working, which increases a sense of support and debriefing around decision making; increasing specialist tools such as AI grading tools; and providing more training for supervisors.

Exercise, wellbeing spaces and social activities were perceived as being positive coping strategies to relieve stress and improve emotional health and wellbeing. “*Better access to exercise in work*,” “*somewhere quiet at work you could go to during the day*”; and social/team building activities “*to create camaraderie*” were all identified as desired resources by participants. Peer-support initiatives were also mentioned:

It would be useful to occasionally hear from officers who have been negatively affected and what they did to overcome that – perhaps as part of monthly wellbeing sessions as guest speakers

There was a desire for regular check-ins with a trained or specialist professional. Participants framed ‘regular’ as ranging from monthly, 6-monthly and annually. Most of these comments included a desire for these check-ins to be face to face and with easier access such as reduced waiting lists.

Face to face annual mental health wellbeing meeting with Occupational Health. At the moment, this is done by way of questionnaire, which does not really reassure staff or identify those who are struggling but may not know they have a problem.

We see someone every 6 months, but it should be the same person and it should be face-to-face

There was support for “*scheduled and mandatory supervision* [which] *would negate the culture/ external factors preventing people from accessing support*” and “remove [the] stigma of having to ask.” Participants suggested that mandatory wellbeing sessions could remove the burden of self-referral and “the pressure to seem strong,” ensure that more specialist support services are not “cast aside by supervision,” and even encourage and improve access to wellbeing services.

There are plenty of options available in my force, but they rely on the individual to reach out to them. I think support should be mandatory and then rejected by the individual if they do not require it. I say this in respect of this role because of the associated stress and trauma that comes with it …. I believe more officers would access help if appointments were made and time was set aside BY THE ORGANISATION [participant’s emphasis]. Otherwise, I feel there will be very little uptake.

## Discussion

The purpose of this paper was to explore the experiences and perceptions of United Kingdom police officers and staff involved in CSAE investigations in relation to existing wellbeing support, barriers to accessing such support, and their desired support services. By building an evidence-based understanding of why police officers and staff do not, or are reluctant to, seek support, and identify what support services they would value, we build on a growing body of work in policing (Hesketh and Tehrani, 2018; College of Policing, 2019; College of Policing, 2020a,b). Clarifying perceptions of support and barriers can equip CSAE investigators to meet the challenges and demands that working in this context brings more effectively.

Our thematic analysis of the data illustrated that many participants were aware of support services, but many also identified barriers which inhibit support-seeking behaviours. These barriers operate on organisational and individual levels and share symbiotic relationships whereby they interact with, create, perpetuate and exacerbate each other. The barriers to seeking mental health support emanated from a lack of trust in their organisations’ approach to wellbeing, workplace culture and practices, all of which were further shaped by stigmatising attitudes towards mental health. Access to, and quality of, existing support services fed into a lack of trust and further exacerbated barriers to seeking mental health support.

### Stigma towards mental ill health

Corrigan (2004, p. 614), in work on how stigma affects mental health care, suggested that stigma can cause two harms. Firstly, it “*may impede treatment*,” and secondly, it can reduce “*self-esteem and rob people of social opportunities*.” Both harms were evident in our findings through participants’ recognition of stigmatising attitudes towards mental health, and towards seeking support for mental health struggles, both of which presented as a permeating dimension of their experience.

Public-stigma, which is the culmination of endorsed prejudice, stereotyping, and labelling felt by individuals (Corrigan, 2004; Wade et al., 2011), was perceived as a powerful barrier which inhibited their help-seeking behaviours and led to under-utilised support services in the current data. These findings echo other research (Corrigan, 2004; Velazquez and Hernandez, 2019; Foley and Massey, 2020; Doyle et al., 2021; Edwards and Kotera, 2021). For example, Vogel et al. (2007) suggested stigma is the most common reason people do not seek counselling for their mental health needs. This is unsurprising given the stigma associated with mental ill health in wider society.

Stigma operates on societal, organisational, and individual levels, all of which interact with each other (Devers et al., 2009) and this emerged from the thematic analysis whereby participants identified the barriers that interacted with and exacerbated each other. Our findings illustrate that feeling stigmatised about one’s mental health can have a ripple effect and translate to fearing being perceived as weak (Parkes et al., 2018; Brown, 2021), lacking capacity to perform the job role, and experiencing a fear of reduced job prospects (Karaffa and Tochkov, 2013; Lee et al., 2020); all are possible consequences of public stigma. In a literature review about police officer and staff responses to exposure to trauma at work, Brown (2021, p. 14) observed that “*fear of stigma and shame permeated throughout the literature*.” This finding highlights not only the prevalence of stigma within policing, but also its power to shape workplace cultures and determine how individuals manage their mental health and wellbeing, illustrating the second harm of stigma, reduced self-esteem and opportunities. As such, stigma, specifically public stigma, can inhibit emotional health and safety because it facilitates a culture which minimises or ridicules experiences and encourages shame and silence, exacerbating emotional and psychological distress (Corrigan, 2004; Seigfried-Spellar, 2018; Foley et al., 2022). Such a pattern can be further compounded when police officers and staff feel that disclosing mental health issues will impact on their current or future job prospects, as was the case for some of these participants.

Overcoming stigma presents a challenge to policing as an organisation, but it is fundamental for creating positive working cultures, which normalise the fact that the CSAE workforce may be impacted by the work at times. Doyle et al. (2021) highlighted that overcoming stigma requires psychoeducation regarding mental health and the development of a mental health agenda. Foley et al. (2022) also recognised the importance of tackling stigma by improving the workplace culture to one where officers feel able, and safe, to talk about their emotions and feelings without fear of negative consequences.

### Organisational approaches to wellbeing and support services

Our analysis showed that participants’ perceptions of how their force approaches the wellbeing of its officers and staff is crucial to building trust and ensuring workers feel valued, and essentially translated to how much they feel cared about and valued. Participants judged their organisation’s approach to wellbeing in terms of its investment in supporting them, evidenced by the availability and quality of support services, and the organisational and workplace culture regarding wellbeing and mental health.

In research examining the experiences and impact of working with sexual offence material, Parkes et al. (2018) recognised that an organisation’s approach to wellbeing sets the tone, culture, and norms regarding wellbeing support needs, and can perpetuate staff perceptions that it is not ‘normal’ to need help. Other research has highlighted the influence of organisational factors, which Tucker (2015) observed were the most important predictor of willingness to seek help. Research by Deschênes et al. (2018, p. 8) into psychological occupational health found that “*organisational context is actually more significant than the operational context*,” and although this work was not carried out with CSAE officers and staff, these findings are in line with ours. Furthermore, the College of Policing (2019) recognises the importance of leadership style and prioritisation of wellbeing in order to build greater resilience and performance in CSAE teams.

Participants reported frustrations with, and a lack of trust in, their managers and supervisors. Low levels of managerial support have been recognised as a challenge for some CSAE investigators (Perez et al., 2010; Powell et al., 2015). The College of Policing (2019, p. 16) recognises that “*Managing employees working with child abuse material requires a high level of leadership and interpersonal skills*” and that supervisors need to be emotionally intelligent and can work collaboratively with officers and staff working in CSAE investigations in order to foster a climate built on trust and respect. This is important because these individuals act as conduits of the wider culture and stigma that may exist regarding mental ill health and access to support services, and Powell et al. (2015) found that CSAE investigators who perceived their supervisors as knowledgeable, caring, and trusting of their team had a positive impact on their wellbeing. However, negative perceptions of, and unsupportive relationships with, senior management, supervisors, and team leaders were identified as barriers in our analysis. Parkes et al. (2018) reported that the attitudes and approaches of individual supervisors were very often a deciding factor as to whether officers sought support or not. Likewise, similar to our findings, they reported examples where people would not seek support as a direct result of interactions with their supervisor.

The importance of positive and supportive relationships with managers and senior officers has been noted in other research. Doyle et al. (2021) highlight that more involvement and training of managers about mental health would help enable them to be more aware of mental health issues earlier, give and signpost to appropriate support and reduce stigma, with their findings suggesting that “*sensitive management*” was important because it can help reduce stigma and fear around seeking support for mental health concerns. Other research highlighted the importance of supervisors who really understood the challenges and impact of working in CSAE investigative teams in supporting them to cope with the role, which increased a sense of wellbeing (Burns et al., 2008; Powell et al., 2015; Parkes et al., 2018). Furthermore, policing guidance on wellbeing acknowledges the importance of a manager’s role in developing a positive model of wellbeing and recommends that managers are well trained in health and wellbeing issues, people skills, difficult conversations, and collaborative working with employees (College of Policing, 2020b).

Investment in support services was perceived by our participants as both a barrier and a reflection of their organisation’s commitment to their wellbeing. Whilst a range of support services were reported as available, with occupational health therapists followed by self-referral to external counselling services being the most available, the findings indicate that only these two services were available to over 50% of the participants, which suggests a potential paucity of service provision for some officers and staff working in CSAE investigations. Furthermore, a low take up of all of the available services was reported, which suggests a situation whereby much of this workforce is without, or does not access, mental health and wellbeing support. Furthermore, participants expressed concern and frustration at the accessibility and quality of the available services. For example, specialist services specifically for CSAE investigators was identified as lacking, which increased participants’ reluctance to seek support, a sentiment that has been found in other criminal justice professionals (Powell et al., 2015).

### Internalised barriers, lack of trust and emotional safety

Our analysis revealed that participants had personal, or internal, barriers to seeking support, which were influenced by organisational, cultural, and stigmatising factors. Corrigan (2004) identifies this as self-stigma, which is an internalisation of public stigma, and relates to a fear of losing respect and credibility (Wade et al., 2011). One consequence of self-stigma is when an individual feels disempowered by their internalisation of the ‘negative’ characteristics and becomes reluctant to seek help (Ben-Zeev et al., 2012). This relates to the second harm caused by stigma identified by Corrigan (2004), which can result in a reduction in self-esteem, which was apparent in some of the participants responses and has been acknowledged in policing guidance (College of Policing, 2019) and found in other research (Lee et al., 2020). It is at the intersection of these factors where participants’ responses, and their decision whether to seek support or not, were formed. Other researchers also recognise the complexities and impacts of organisational factors and individual reactions (Parkes et al., 2018; Doyle et al., 2021). There was a sense of demoralisation regarding much of the available support and a reluctance to access it to seek help.

Omnipresent throughout our analysis was a palpable sense of participants’ lack of trust towards their organisation and managers, which was woven through all the barriers already discussed. Participants did not feel safe disclosing struggles with mental health and wellbeing. Participants were also uncomfortable using some services and strategies, such as wellbeing days, for fear of judgement, and lack of confidentiality and concern about the consequences of disclosing mental health struggles such as fear of being perceived as weak, incapable, or a burden were reported in other research as well as in ours (Deschênes et al., 2018; Seigfried-Spellar, 2018; Lee et al., 2020). Some participants reported negative experiences of using services and adopted a ‘going it alone’ approach to protect their mental health. Similar experiences were reported by Parkes et al. (2018), whereby participants in their research felt that they had to hide any difficulties they were having around their mental health due to cultural expectations and pressure that they should be able to cope, and a fear of being perceived as weak or failing. Our analysis showed that the lack of work-based support for participants’ wellbeing often exacerbated their struggles with their emotional and mental health. Such findings resonate with those of Deschênes et al. (2018, p. 8), who argued “that a lack of emotional support at work is more likely to be harmful to psychological health than the operational tasks police officers carry out within the performance of their duties.”

In sum, our analysis reveals a sense of a lack of emotional safety amongst participants when it came to seeking mental health support within their organisation. Lack of emotional safety was the result of the interplay between dynamic factors including organisational and workplace cultures and approaches to mental health and well-being, stigma and judgements surrounding mental ill health and wellbeing, and how an individual makes sense of and responds to the environment created by these factors.

### Desired support resources

The supportive resource participants identified as most desiring for their workplaces was ‘a workplace culture that explicitly values and prioritises the emotional health and wellbeing of the workforce’. This and other research recognise the importance of workplace culture, and that without a positive and supportive culture, barriers to seeking mental health support remain (Evans et al., 2013; Sollie et al., 2017; Burns and Buchanan, 2020; Brown, 2021; Tapson et al., 2021). Participants also identified social support as a coping mechanism and tool to build individual and team resilience. The fact that social support was a highly desired resource aligns with other research (Tomyn et al., 2015; MacEachern et al., 2019). Exercise, as a form of stress release, was also identified by some participants in our research as desirable, a finding also echoed in other research (Lee et al., 2020). However, Bourke and Craun (2014) found that exercise was not a particularly effective tool in combating secondary traumatic stress in CSAE investigators, so further research would be useful. Participants wanted immediate and on-going access to support services, and in alignment with other research into support for high-risk roles (Doyle et al., 2021), they expressed the desire for specialist professionals who understand the particular nature of their work as CSAE investigators.

### What good support looks like

What participants believed good support should look like was summed up as a “*workplace culture which explicitly values and prioritises the emotional health and wellbeing of the workforce, not just playing lip service to the idea without actually engaging with basic staff needs.*” Therefore, to remove barriers to wellbeing support for CSAE investigators, police organisations need to introduce policies and practices which eliminates the stigma attached to mental ill health and talking about one’s struggles. This is likely to require:

- Emotional and psychological health and wellbeing to be reframed as a health and safety concern and elevated to become Authorised Professional Practice endorsed by the College of Policing.
- A re-set of expectations around mental health, which normalises the need for emotional and psychological support to help manage the impacts of this work.
- A coherent and consistent continuum of care, beginning at recruitment, available regularly for the duration of the role, and an offer of post-role support.
- Sensitive and well trained emotionally intelligent managers and supervisor who have a specialised knowledge and understanding of what working in CSAE investigations involves, and the impact that can have.
- Improved workplace practices that facilitate time and space to process the impact of the work for teams and individuals.
- A range of good quality and appropriate support services which includes professionals with a specialist understanding of CSAE.
- Consistent provision across and within forces.

Alongside the provision of effective support as described above, attention could also be directed at the implementation of work-based training to help promote the development of factors known to be associated with wellbeing in the workplace. In particular, the role of emotional intelligence (e.g., Brunetto et al., 2012), resilience (e.g., Andersen et al., 2015) and stress management. (e.g., Richardson and Rothstein, 2008; Acquadro-Maran et al., 2015) have all been shown to be effective in improving wellbeing in police settings. For example, Romosiou et al. (2019) investigated the effectiveness of a group programme designed to enhance emotional intelligence, empathy, resilience and stress management skills in police officers in Greece. Their results showed significant improvement across all factors, which were three months after the end of the intervention. Similarly, but in a United Kingdom setting, Hesketh et al. (2018), found support for the use of resilience training programmes for improving workforce well-being by addressing sources of stress and educating the workforce in how to deal with stressors.

## Study strengths and limitations

The current study has a number of strengths. To the best of our knowledge, it is the first study to explore the views of police officers and staff specifically working in CSAE investigation roles around work-based wellbeing support and barriers to access. Furthermore, the study includes all four United Kingdom nations making it the first national study of its kind. In addition, the sample size was large for a study of this kind, with hundreds of participants not only completing quantitative measures but also writing text comments. Lastly, the findings have clear implications for policy and practice.

That said, like all research, this study has a number of limitations. First, the sample may not be representative of all CSAE staff, as it may be that those who were motivated to take part in the survey, and provide open-text responses, did so because of their particularly negative views or experiences of work-based wellbeing support. However, whilst in the minority, there were also participants who reported positively on the wellbeing support provision in their organisation.

The sample is also of potentially limited generalizability, in that there was a lack of ethnic diversity in our sample, with the vast majority of participants identifying as White. However, this sample does reflect the current ethnic makeup of the United Kingdom police, with White officers making up 92.4% of the workforce in England and Wales as of 31 March 2021 (Police Workforce: England and Wales, 2021), 98.5% in Scotland (Police Scotland, 2022) and 99.4% in Northern Ireland (Police Service of Northern Ireland, 2022). Also, most participants were from forces in England, with fewer responses from Wales, Scotland and Northern Ireland. When compared against current police numbers, Scotland was under-represented in our sample (6.5% of our sample versus 10.5% of total police workforce), England and Wales was over-represented (89.3% versus 85.6%) and Northern Ireland was perfectly represented (4.2% for both). Whilst this pattern matches the relative size of the force in each country (Allen and Mansfield, 2022), it nonetheless could be that this pattern obscures particular dynamics in subsets of the sample. Lastly, more women than men responded, which does not reflect the makeup of the police population. As of March 2021, police were comprised of 32.4% female officers in England and Wales (Police Workforce: England and Wales, 2021); 32.0% in Scotland (Police Scotland, 2022) and 31.4% in Northern Ireland (Police Service of Northern Ireland, 2022). The overrepresentation of women in the current sample may reflect a difference in attitudes and willingness to discuss issues around mental health and wellbeing. However, quantitative analyses of this sample showed no significant difference between men and women on any mental health outcomes, suggesting that the current results remain nonetheless informative (Conway et al., 2023). In sum, these limitations suggest that caution should be exerted generalising from the results of this study. That said, the strong alignment of the results of this work with past work suggests that the current results may indeed reflect broader trends in CSAE personnel and police more generally.

## Conclusion

Working in CSAE investigations inherently involves risks to the emotional and psychological wellbeing of the officers and staff involved. Our analysis revealed a fundamental sense of a lack of emotional safety when it came to seeking mental health support within policing. This was the result of the interplay of dynamic factors regarding wellbeing including organisational and workplace cultures, stigma and judgements, and individual responses to the environment created by these factors. In order to protect the wellbeing of police officers and staff, and prevent poor wellbeing, there is a need to explicitly recognise and accept these risks. Reframing and elevating wellbeing as a health and safety concern is key to developing an effective and consistent approach to protecting and maintaining good mental health. To reduce barriers to seeking support, there needs to be a normalisation of the impacts and struggles caused by working in this space, and an elimination of stigma regarding mental ill health within policing. Such an approach needs to draw on all the good practice that is ongoing within policing and other sectors and be embedded in, and available to, all forces.

## Data availability statement

The datasets presented in this study can be found in online repositories. The names of the repository/repositories and accession number(s) can be found at: https://osf.io/5ru7h/?view_only=5bf8c668682f4a4783d439456eb2a397.

## Ethics statement

The studies involving human participants were reviewed and approved by University of Portsmouth Faculty of Creative and Cultural Industries Research Ethics Committee, Reference Number CCI-FEthC 2022-003. The patients/participants provided their written informed consent to participate in this study.

## Author contributions

TR, SL, PC, and PL study conception and design. TR, PC, and SB data collection. TR, SL, and PC analysis and interpretation of results. TR and SL draft manuscript preparation. All authors reviewed the results and approved the final version of the manuscript.

## Funding

This study was supported by The Dawes Trust, grant number GXK/173783/00001.

## Conflict of interest

The authors declare that the research was conducted in the absence of any commercial or financial relationships that could be construed as a potential conflict of interest.

## Publisher’s note

All claims expressed in this article are solely those of the authors and do not necessarily represent those of their affiliated organizations, or those of the publisher, the editors and the reviewers. Any product that may be evaluated in this article, or claim that may be made by its manufacturer, is not guaranteed or endorsed by the publisher.

## Footnotes

^1^Many terms and acronyms are used within this space. Where we are reporting on research that uses other terms, we use child sexual abuse and exploitation/CSAE as an umbrella term to create consistency.

^2^This is used as an umbrella term to encapsulate all other terms used to describe child sexual abuse materials.

^3^Operation Hydrant delivers the national policing response to non-recent CSAE investigations which relate to offences in an institutional setting and/or concern persons of ‘public prominence’ (https://www.npcc.police.uk). The total number of alleged suspects referred to Operation Hydrant is 9,367, and the total number of victims/survivors is 12,701 since 2014 (National Police Chiefs’ Council, 2022).

^4^Characterised by a sense of extreme exhaustion, disengagement from work, reduced role effectiveness (Brady, 2017).

^5^Characterised be a lack of “*capacity or … interest in bearing the suffering of others*” (Figley, 2002, p. 1434).

^6^Characterised by three components: a betrayal of what is morally right; by someone who holds legitimate authority; in a high-stakes situation (Shay, 2014, p. 183).

^7^A response to exposure to a traumatic stressor that results in a re-experiencing of the trauma, avoidance of similar stimuli, increased arousal, vigilance and numbing behaviours (Breslau, 2009).

^8^A natural response to “the stress resulting from helping or wanting to help a traumatised or suffering person” (Figley, 1995, p. 10), often associated with indirect exposure to trauma.

^9^Stigma is “a negative stereotype and attitude leading to undesirable experiences or opinions” (Velazquez and Hernandez, 2019, p. 714).

^10^A write-up of the survey results outside of the scope of the present paper can be found in Conway et al. (2023).

^11^This refers to “the explicit or surface meanings of the data and the analyst is not looking for anything beyond what a participant has said or what has been written.” (Braun and Clarke, 2006, p. 84).
